# Exchange transfusion for neonate with haemolytic uremic syndrome

**DOI:** 10.1186/s40064-016-1667-x

**Published:** 2016-01-20

**Authors:** Bedangshu Saikia, Neetu Vashisht, Neeraj Gupta, Archna Sharma

**Affiliations:** Department of Pediatrics and Neonatology, St Stephens Hospital, Tis Hazari, New Delhi, 110054 India; Department of Pathology, St Stephens Hospital, Tis Hazari, New Delhi, 110054 India

**Keywords:** Birth asphyxia, Meconium aspiration, Microangiopathic haemolytic anaemia (MHA), Neonatal HUS

## Abstract

**Introduction:**

Haemolytic uremic syndrome (HUS) is one of the most common causes of acute renal failure in children but it is uncommon in newborns. To our knowledge only five cases have been reported so far (probably underreported). The known modalities of treatment include transfusion of plasma and plasmapheresis. We report a case of neonatal HUS for whom we performed an exchange transfusion to good effect.

**Case description:**

A term vaginally born baby, meconium stained and floppy at birth presented with severe anaemia in the first few hours of life. The baby later on developed renal failure and blood picture was suggestive of severe thrombocytopenia and microangiopathic haemolytic anaemia. No extra renal manifestations of birth asphyxia were noted. A double volume exchange transfusion was performed relatively early and subsequently platelet and haemoglobin stabilised and renal failure improved.

**Discussion and evaluation:**

The clinical impression in this case was convincing of neonatal HUS, likely attributable to birth asphyxia but needs to be differentiated from disseminated intravascular coagulation (DIC) and thrombotic thrombocytopenic purpura (TTP). The coagulation profile is usually normal in HUS but it is abnormal in DIC, whereas in TTP one would find hyperbilirubinemia, increased creatinine, haemolysis etc. TTP is rare but not very uncommon in infancy. Congenital TTP is attributed to an inherent deficiency of ADAMTS-13, which is a vWF-cleaving metalloprotease. Irrespective of the etiology of HUS in our case, a dramatic response was observed with exchange transfusion. Transfusion of fresh frozen plasma (FFP) and plasmapheresis are known treatment modalities. FFP replaces the missing or altered complement factors and plasmapheresis removes antibodies, immune complexes and toxins. An exchange transfusion combines both these functions.

**Conclusions:**

In the absence of facilities for plasmapheresis, exchange transfusion is a good alternative.

## Introduction

Neonatal haemolytic uremic syndrome (HUS) is characterised by the triad of microangiopathic haemolytic anaemia (MHA), thrombocytopenia and renal failure (Corrigan and Boineau 2001). It is rare in newborns; to be very precise probably under-reported. To our knowledge, only five cases of neonatal HUS have been reported to-date; three were found to be associated with birth asphyxia (Biran et al. 2007), one was associated with transplacental transmission of *E. coli* O157 (Ulinski et al. 2005) and another related to intracellular vitamin B12 deficiency (Kind et al. 2002).

We describe the case of a neonate with HUS secondary to birth asphyxia presenting as severe anaemia, as early as 4 h of life. The anaemia was refractory to red cell transfusions. The haematological and renal profile improved following a double volume exchange transfusion done at 16 h of life. Plasmapheresis and plasma infusions have been used previously to treat HUS. Exchange transfusion essentially provides plasma exchange besides augmenting the haemoglobin levels. Arguably this is the first report of neonatal HUS treated with exchange transfusion.

## Case description

A three kilogram baby boy was born vaginally at 38 weeks of gestation to a primiparous mother. The baby was meconium stained and not vigorous at birth. Endotracheal intubation and positive pressure ventilation were needed for resuscitation. He was extubated at 3 min of life, after establishment of adequate respiratory efforts.

Haemoglobin at birth was 18.3 g/dL and the platelet count was 1.62 × 10^9^/L. A blood smear examination done at 1 h of life was normal (Table 1). At around 4 h of life, marked pallor was noted. Respiratory distress (respiratory rate 70/minute), subcostal retractions and falling saturations necessitated initiation of bubble CPAP. CPAP of 8 cm of water and FiO_2_ of 50 % were needed to maintain oxygen saturations above 90 %. Falling blood pressure and other markers (clinical and biochemical) of hemodynamic compromise prompted fluid resuscitation followed by ionotropes. Dobutamine was started to augment the falling cardiac output. Subsequently, oligo-anuria was also noted to have set in.

**Table 1:** Table of investigations

Parameters (in SI units)	Age of the index case (in hours, in the first 72 h) and investigations done							
	1 h of life	4 h of life	7 h of life	14 h of life	29 h of life	53 h of life	65 h of life	Day 4 of life
Total leucocyte counts, TLC (per μL)	26,100	12,200	25,200	6500	6700	7500		11,900
Hemoglobin (gm/dL)	18.3	7.7	6.4	12.1	13.1	12.9		12.7
Hematocrit (in %)	57.8	23.1	20	36.4	39.3	39.1		38.4
Platelet count (per μL)	162,000	43,000	64,000	70,000	80,000	89,000		108,000
Reticulocyte count (in %)		5.6	7.8					
Direct coomb’s test			Negative					
Blood urea nitrogen (mg/dL)			12.0	40.3	18.7	16.2	10.6	4.4
Creatinine (mg/dL)			1.3	1.6	1.2	0.8	0.6	0.5
Prothrombin time (s)		14.8						
Partial thromboplastin time (s)		28.7						
INR (international normalized ratio)		1.05						
Sodium (mEq/L)			145.3	137.1	145.0	136.4	138.0	143.3
Potassium (mEq/L)			4.3	4.1	3.84	3.60	5.11	5.0
Serum total calcium (mg/dL)			9.7	6.1	6.1	11.3	7.0	9.7
Serum total bilirubin (mg/dL)		1.72	8.70					
Serum indirect bilirubin (mg/dL)		1.36	7.91					
Creatinine phosphokinase (U/L)					1414			
CK–MB (U/L)					96			
pH	7.181	7.153	7.198		7.419			
CO_2_ (mmHg)	43.3	21.4	28		46.3			
HCO_3_ (mmol/L)	15.6	7.2	15.6		29.5			
Lactate (mg/dL)	8.7	22.5	6.8		1.5			
Schistocytes in peripheral blood smear		Present (Fig. 1)						

**Fig. 1:**
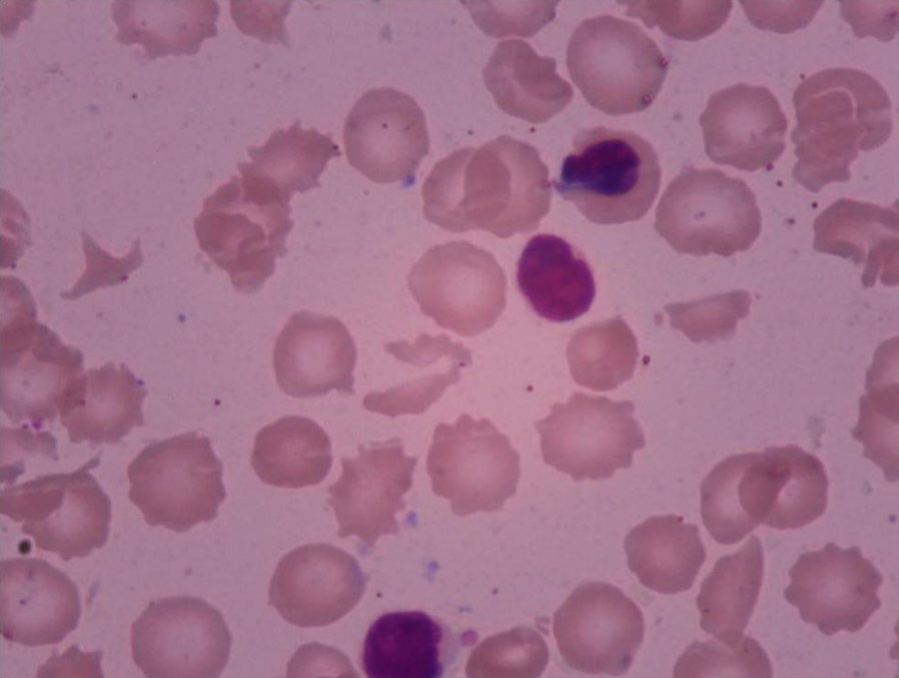
Peripheral smear showing schistocytes classical of microangiopathic haemolytic anaemia

The results of the investigations at 4 h are detailed in the Table 1. The haemoglobin had fallen to 7.7 g/dL and platelets had decreased to 43,000/mm^3^. Peripheral smear showed fragmented RBCs and schistocytes suggestive of MHA (Fig. 1). The baby was switched on to intermittent positive pressure ventilation at 5 h of life in view of worsening respiratory distress. Chest X-ray did not reveal any lung parenchymal abnormality. Packed cells (45 mL) were transfused at 6 h of life, followed by a second transfusion (45 mL) as the haemoglobin did not rise satisfactorily following transfusion of the first aliquot. Haemoglobin remained low (6.7 g/dL) even after two transfusions at 7 h of life. The reticulocyte count was 7.8 %. Blood urea nitrogen was 40.3 mg/dL and serum creatinine was 1.6 mg/dL at 14 h of life. Prothrombin time, partial thromboplastin time and FDP were normal. There was no haematuria and proteinuria or bleeding from peripheral cannulation sites. Ultrasound examination of cranium and abdomen were normal. The blood group of the baby was compatible with the mother’s and direct Coombs test was negative. There was no family history of atypical HUS. There were no extra-renal manifestations of birth asphyxia.

On account of the falling haemoglobin which continued despite packed cell transfusions, the evidence of red blood cell fragmentocytosis, deranged renal functions and the normal coagulation screen, a diagnosis of neonatal HUS was made. 90 mL of fresh plasma was transfused. A double volume exchange transfusion with whole blood cross-matched with both baby and mother was performed using 480 mL of group A Rh positive blood. Following the exchange transfusion, haemoglobin increased to 13.1 gm/dL, platelet count increased to 80,000/mm^3^, BUN decreased to 18.7 mg/dL, serum creatinine fell to 1.2 mg/dL and urine output improved gradually as well. No further blood transfusions or renal replacement therapy were needed. The baby was uneventfully extubated on day 4 of life and transferred to ward on day 5 of life. The baby is now 9 months old and doing well in follow up. He has had no significant illness or hospitalisation since discharge from our hospital.

## Discussion and evaluation

The clinical picture in this case was suggestive of neonatal HUS, most likely attributable to birth asphyxia. Drastic falls in haemoglobin in absence of bleeding, as occurred in our neonate and fragmentocytes seen on the smear were suggestive of HUS. Serum haptoglobin levels and LDH are used as axillary tools in diagnosing intravascular hemolysis, but they are not reliable in a newborn due to inherent hepatic immaturity and these tests were not done in our case.

HUS needs to be differentiated from disseminated intravascular coagulation (DIC) and thrombotic thrombocytopenic purpura (TTP). There are no tests that can definitely differentiate HUS from DIC except that the coagulation profile is usually normal in HUS but it is abnormal in DIC. The fact that the child did not suffer any other MHA/TMA episode supports the idea that he does not have congenital TTP as the rate of recurrence is very high with congenital TTP; more than 60 % of patients have their relapses within the first year and about 90 % relapses within the first 4 years. (Hovinga et al. 2010).

TTP can also present with similar symptomatology and associated thrombocytopenia, hyperbilirubinemia, increased creatinine, hemolysis etc. (Moake 2002). Though rare, TTP is not as uncommon in infancy as previously thought. The congenital presentation of TTP is attributed to an inherent deficiency of vWF-cleaving metalloprotease (ADAMTS-13) (Moake 2002). However, measurement of ADAMTS-13 in newborns is highly laboratory-dependent and also the fact that it is a costly investigation to obtain.

Non-immune thrombocytopenia in association with microangiopathic haemolytic anaemia can also occur in Kasabach Merritt syndrome and renal vein thrombosis. There was no suggestion of these in our neonate.

Three cases of neonatal HUS following asphyxia have been reported till date (Biran et al. 2007). They also presented with a features of anaemia, thrombocytopenia and schistocytes on peripheral smear. Renal failure was present in all three cases. Two cases were treated with peritoneal dialysis while the other showed spontaneous improvement in renal parameters. The earliest onset was at 12 h. Biran et al. (2007) hypothesised that “endothelial damage that occurs in birth asphyxia leads to a vicious cycle of consumption of platelets and plasma factors involved in hemostasis and fibrinolysis (complement factors H/I and ADAMTS13)”. Complement factor H/I deficiency is known to cause excessive activation of the alternative complement pathway causing injury to the capillary endothelial cells and subsequent symptoms of HUS (Caprioli et al. 2001). ADAMTS13 is a metalloprotease enzyme which is needed to cleave the large multimers of vWF (von Willebrand factor) into smaller inert fractions. In the absence of this enzyme, the uncleaved large multimers induce platelet adhesion and aggregation at sites of endothelial injury leading to platelet rich thrombi in the end organs (Furlan and Lämmle 2000). Besides consumption of these factors, congenital deficiency of ADAMTS can also precipitate HUS. We did not investigate for these deficiencies and it is possible that these deficiencies coexisted in our case.

Whatever the exact etiology of HUS in this case (whether secondary to alternate complement pathway activation or paucity of ADAMTS13) it responded dramatically to exchange transfusion (Licht et al. 2005). Traditionally, transfusion of fresh frozen plasma (FFP) and plasmapheresis have been used in treatment of HUS irrespective of the exact etiopathogenesis (Scully et al. 2012). Currently, the monoclonal antibody eculizumab is being used in some centres for the treatment of refractory TTP (Chapin et al. 2012); Besbas et al. (2013) used this therapy successfully to treat an infant with recurrent disease. Treatment with eculizumab is expensive and takes time for its effects though. FFP acts by replacing the missing or altered complement factor H/I or ADAMTS13 enzyme. Plasmapheresis removes antibodies (which consume the ADAMTS13) as also the immune complexes and toxins but it is not useful in the congenital forms of HUS. An exchange transfusion combines both these functions of removing antibodies and replenishing the deficient or altered proteins (Coppo et al. 2010). In the absence of facilities for plasmapheresis, exchange transfusion is a good alternative. Exchange transfusions are performed in most neonatal units usually in the context of neonatal hyperbilirubinemia. Neonatal HUS may be a new indication for this procedure.

## Conclusions

Neonatal HUS is a very rare complication and even rarer associated with birth asphyxia. Early differentiation from similar presentation and diagnosis is important in terms of further planning and outcome. Exchange transfusion may be a viable alternative to plasmapheresis in resource limited settings.

## References

[CR1] Besbas N, Gulhan B, Karpman D (2013). Neonatal onset atypical hemolytic uremic syndrome successfully treated with eculizumab. Pediatr Nephrol.

[CR2] Biran V, Fau S, Jamal T (2007). Perinatal asphyxia may present with features of neonatal atypical hemolytic uremic syndrome. Pediatr Nephrol.

[CR3] Caprioli J, Bettinaglio P, Zipfel PF, Italian Registry of Familial and Recurrent HUS/TTP (2001). The molecular basis of familial hemolytic uremic syndrome: mutation analysis of factor H gene reveals a hot spot in short consensus repeat 20. J Am Soc Nephrol.

[CR4] Chapin J, Weksler B, Magro C, Laurence J (2012). Eculizumab in the treatment of refractory idiopathic thrombotic thrombocytopenic purpura. Br J Haematol.

[CR5] Coppo P, Schwarzinger M, Buffet M (2010). Predictive features of severe acquired ADAMTS13 deficiency in idiopathic thrombotic microangiopathies: the French TMA reference center experience. PLoS ONE.

[CR6] Corrigan JJ, Boineau FG (2001). Hemolytic-uremic syndrome. Pediatr Rev.

[CR7] Furlan M, Lämmle B (2000). Haemolytic-uraemic syndrome and thrombotic thrombocytopenic purpura—new insights into underlying biochemical mechanisms. Nephrol Dial Transplant.

[CR8] Hovinga JA, Vesely SK, Terrell DR (2010). Survival and relapse in patients with thrombotic thrombocytopenic purpura. Blood.

[CR9] Kind T, Levy J, Lee M (2002). Cobalamin C disease presenting as hemolytic-uremic syndrome in the neonatal period. J Pediatr Hematol Oncol.

[CR10] Licht C, Weyersberg A, Heinen S (2005). Successful plasma therapy for atypical hemolytic uremic syndrome caused by factor H deficiency owing to a novel mutation in the complement cofactor protein domain 15. Am J Kidney Dis.

[CR11] Moake JL (2002). Thrombotic microangiopathies. N Engl J Med.

[CR12] Scully M, Hunt BJ, Benjamin S, on behalf of British Committee for Standards in Haematology (2012). Guidelines on the diagnosis and management of thrombotic thrombocytopenic purpura and other thrombotic microangiopathies. Br J Haematol.

[CR13] Ulinski T, Lervat C, Ranchin B (2005). Neonatal hemolytic uremic syndrome after mother-to-child transmission of *Escherichia coli* O157. Pediatr Nephrol.

